# Spin Hall magnetoresistance and spin Seebeck effect in Pt |CoCr_2_O_4_ heterostructures

**DOI:** 10.1080/14686996.2025.2457320

**Published:** 2025-02-03

**Authors:** Aisha Aqeel, Matthias Kronseder, Nynke Vlietstra, Hans Huebl, Jeroen A. Heuver, Beatriz Noheda, Javier Herrero-Martín, Eric Pellegrin, Hari B. Vasili, Maxim Mostovoy, Christian Back

**Affiliations:** a School of Natural Sciences, Technical University of Munich, Garching, Germany; b Munich Center for Quantum Science and Technology (MCQST), Munich, Germany; c Department of Physics, Regensburg University, Regensburg, Germany; d Institute of Physics, University of Augsburg, Augsburg, Germany; e Walther-MeiBner-Institut, Bayerische Akademie der Wissenschaften, Garching, Germany; f Zernike Institute for Advanced Materials, University of Groningen, Groningen, The Netherlands; g ALBA Synchrotron Light Source, Cerdanyola del Valles, Barcelona, Spain

**Keywords:** Spin Hall effect, magnetoresistance, magnetic proximity effects, noncollinear magnet, spin spiral, conicity

## Abstract

This study delves into spin current-induced phenomena, such as spin-Hall magnetoresistance and the spin Seebeck effect within Pt films deposited on a noncollinear magnet, CoCr${\;}_{2}$O${\;}_{4}$ (CCO), particularly at low temperatures. Detailed investigation of the angular dependencies of spin Hall magnetoresistance (SMR) and spin Seebeck effect (SSE) was carried out. The temperature-dependent behavior of both SMR and SSE signals exhibits a discernible variation correlated with different magnetic phases of CCO. To distinguish the contributions arising from magnetic proximity effects, we conducted X-ray magnetic dichroism (XMCD) at the Pt-M${\;}_{3}$ edge. XMCD data from Pt/CCO heterostructures suggest that any magnetic moment associated with Pt, if present, is below the detection limit. This supports the notion that the observed signals primarily stem from SMR and SSE. This study offers insights into spin-current-driven phenomena, paving the way for potential spintronic applications.

## IMPACT STATEMENT

This study uniquely differentiates non-collinear magnetic phases in CoCr₂O₄ using all-electrical SMR and SSE, validated by excluding proximity-induced magnetization via XMCD, advancing spintronic detection techniques.

## Graphical abstract

**Figure:**
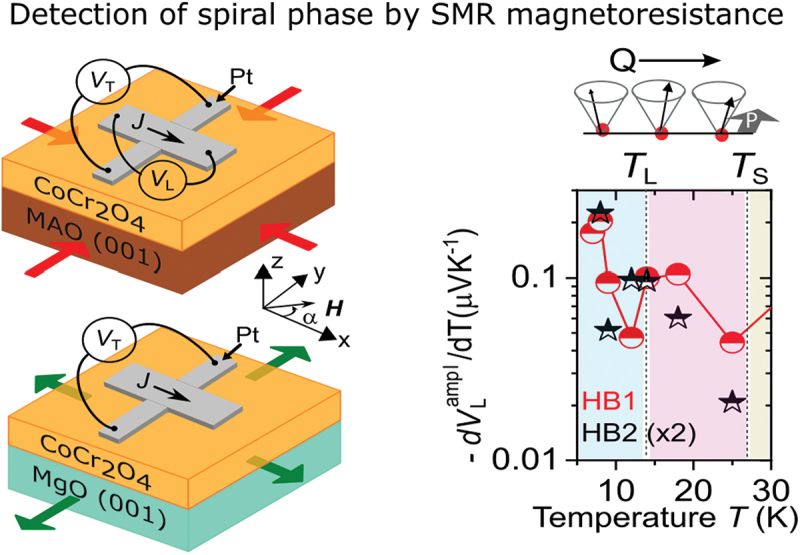

## 1. Introduction

Pure spin currents offer the potential for more energy-efficient spintronics and can be generated not only in magnetic insulators but also in normal metals with large spin-orbit coupling. In normal metals, pure spin currents cause a spin accumulation at their surfaces, capable of interacting with the magnetization of an adjoint ferromagnetic insulator. This interaction leads to a resistance change in Pt, depending on the relative magnetization direction of the adjacent magnet. A phenomenon recognized as the spin Hall magnetoresistance (SMR) [1,2]. As a consequence, the SMR can be anticipated as an avenue for electrical probe for sensing magnetization direction and the presence of an ordered magnetic phase [3] in ferromagnetic insulators. This capability has been explored across diverse magnetic systems, including collinear ferrimagnetic insulators such as Yttrium iron garnet (Y${\;}_{3}$Fe${\;}_{5}$O${\;}_{12}$) [2, I], antiferromagnetic insulators e.g. NiO [4], and α-Fe${\;}_{2}$O${\;}_{3}$ [5] and noncollinear magnets like Cu${\;}_{2}$OSeO${\;}_{3}$ [3,6] and CoCr${\;}_{2}$O${\;}_{4}$ [7]. In the spin Seebeck effect (SSE), spin currents carried by thermal excitations of the magnetic order, like spin waves or magnons, are transferred into a metallic electrode and converted within the metal to an orthogonal charge current, which is typically detected as electrical potential difference using open-circuit conditions. The conversion of spin current to charge current in SSE is intricate, and while it doesn’t directly reveal the surface magnetization of a magnetic insulator, it can serve to delineate variations in surface magnetization, as shown in Refs [7,8].

**Figure 1.**
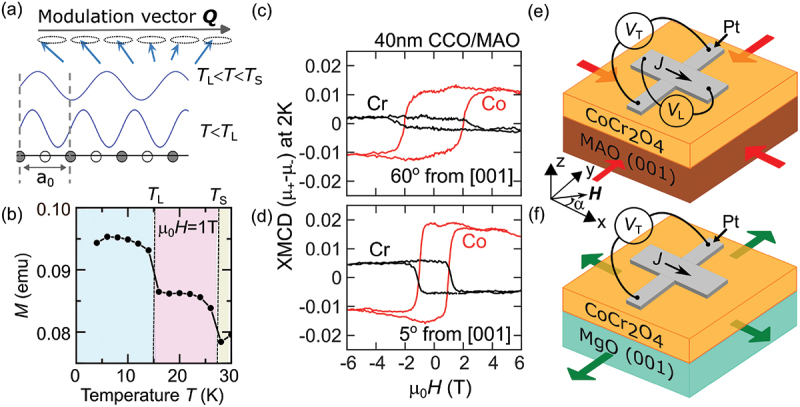
(a) Impression of the spiral magnetic order in CoCr${\;}_{2}$O${\;}_{4}$ (CCO) that transforms from an incommensurate to a commensurate spiral order below $T_{L}$. (b) Temperature dependence of the zero-field cooled magnetization measured in a 1T applied magnetic field for CCO powder used as source material for the thin-film deposition, where $T_{s}$ and $T_{L}$ denote the transition temperatures for spin-spiral and spin lock-in magnetic phases. where $T_{S}$ and $T_{L}$ denote the transition temperatures for spin-spiral and spin lock-in magnetic phases. (c) and (d) The X-ray magnetic circular dichroism (XMCD) response as a function of the applied magnetic field. Measurements were taken at angles of 60${\;}^{\circ }$ (at grazing incidence 30${\;}^{\circ }$ away from the film plane) and 5${\;}^{\circ }$ (normal incidence) relative to the out-of-plane [001] axis. The XMCD signal corresponds to photon energies at the Co and Cr L${\;}_{3}$ edges. (e) and (f) schematic representation of 5 nm thick Pt Hall bars on CCO thin films deposited on MAO substrates under compressive strain (red arrows) and MgO substrates under tensile strain (green arrows), respectively. The coordinate system introduces the rotation angle α in the x-y plane between the applied magnetic field H and the applied current density J in the Pt bar.

Among magnetic insulators, CoCr${\;}_{2}$O${\;}_{4}$ (CCO) is unique as it is a type II multiferroic [9–11], that exhibits ferroelectricity and a remnant magnetic moment in the same phase due to significant magnetoelectric coupling [12]. CCO has a normal spinel crystal structure with Co${\;}^{2+}$ and Cr${\;}^{3+}$ magnetic ions distributed within tetrahedrally and octahedrally coordinated sites, respectively. CCO belongs to the space group Fd-3 m (No. 227) and the $O_{h}$ point group. The Co and Cr magnetic sublattices are antiferromagnetically coupled, with the Co sublattice exhibiting larger moments at all temperatures [13] with no compensation point. CCO is a well-characterized ferrimagnet with $T_{c}$ = 95 K and with two additional magnetic phases known as spin-spiral and spin lock-in magnetic phases [14]. In the spin-spiral magnetic phase, below $T_{S}\approx$ 27 K, an additional long-range spin-spiral with cycloidal spin modulation occurs [12]. In the spin lock-in magnetic phase (below $T_{L}\approx$ 15 K), the period of the spiral becomes commensurate with the lattice periodicity [12,15–17] (see Figure 1(a)).

A spin lock-in phase in thin films has stirred some debate, primarily because these films incorporate significant contributions from paramagnetic impurities in the substrate, complicating characterization via conventional magnetometry techniques. A recent investigation of CCO films illustrated substantial alterations in magnetic anisotropy attributed to strain [18]. Windsor et al. [19] employed x-ray techniques to investigate the magnetic behavior of a strained, [110] oriented CoCr${\;}_{2}$O${\;}_{4}$ film. Their findings revealed the emergence of only the spin spiral phase below the transition temperature $T_{S}$, coinciding with the ordering temperature observed in the bulk CCO samples.

All-electric magnetization detectors based on spin currents are key elements in spintronic devices. A promising candidate is SMR [1,2] in heterostructures comprising metallic electrodes and magnetic insulators. The magnitude of SMR in e.g. collinear magnets is roughly approximated by the total in-plane magnetic moment density present at the surface. SMR has been widely used as a magnetization detector to monitor changes in surface magnetization across various magnetic systems. However, in this study, we focus on systematically investigating and detecting magnetic phases characterized by periodic spiral modulations. Such complex spin structures, like magnetic spiral phases, are not only challenging to detect but can also be significantly influenced by strain effects in thin films compared to their bulk counterparts. This raises a critical and intriguing question: can SMR serve as an all-electric sensor for detecting magnetic spiral phases, particularly in frustrated magnets where these spirals exhibit an exceptionally small spiral period? Addressing this challenge is of great importance for advancing the understanding and application of these intricate magnetic configurations in spintronic technologies. Presently, only one study by Aqeel et al. [7], demonstrates SMR and spin Seebeck effect (SSE) in the Pt/CCO heterostructures. The authors systematically related the observed SMR and SSE signal amplitudes to different magnetic phases of CCO. Our work explicitly disentangles the contributions of proximity-induced magnetization in Pt [20] using XMCD measurements. This approach provides a more direct assessment of the intrinsic magnetic and spin transport properties in Pt/CCO heterostructures.

This paper systematically investigates the SMR and spin Seebeck effect (SSE) in strained CCO thin films. We determine the magnetic transitions from SMR and SSE measurements and compare them to the magnetic properties of CCO powder. We also explore the thickness dependence of SMR and SSE signals in these films. We used the X-ray dichroism technique (XMCD) to eliminate the contributions from proximity effects in Pt on CCO films. XMCD – being an atomically selective magnetization probe has been used to assess the presence or absence of magnetic proximity effect at the interface of metallic electrodes with other spinel oxides, such as CoFe${\;}_{2}$O${\;}_{4}$ [21,22] and NiFe${\;}_{2}$O${\;}_{4}$ [23].

## 2. Materials and methods

### 2.1. Sample preparation and characterization

The magnetization of the CoCr${\;}_{2}$O${\;}_{4}$ (CCO) powder used as source material for film deposition was measured using a SQUID magnetometer, shown in Figure 1(b). The temperature dependence of the magnetization clearly shows the spin-spiral and spin lock-in magnetic transitions at $T_{S}$ and $T_{L}$, respectively. in the CCO power. The CoCr${\;}_{2}$O${\;}_{4}$ (CCO) films with thicknesses ranging from 20 to 80 nm were grown on MgO and MgAl${\;}_{2}$O${\;}_{4}$ (MAO) substrates using pulsed laser deposition [18]. The spinel crystal structure of CCO led to a lattice mismatch, inducing a tensile in-plane strain in CCO/MgO films and a significant compressive in-plane strain in CCO/MAO films. The compressive strain in the CCO film enhances magnetic anisotropy along the out-of-plane direction. Figures 1(c,d) illustrate the hysteresis loops obtained by XMCD for a 40 nm thick CCO/MAO film in the in-plane and out-of-plane directions, respectively.

Pt Hall bar structures (5 nm thick, 20 μm wide, with a contact separation of 800 μm) were patterned on both CCO/MgO and CCO/MAO films. We used two different substrates (MAO and MgO) for CCO films to examine how strain impacts the magnetic and spin transport properties of the films. Unless stated otherwise, all the data presented in this experimental section were obtained from a 40 nm thick CCO/MAO sample. Furthermore, two Hall bar devices (HB1 and HB2) were fabricated on the CCO/MAO sample to compare the results of different areas of the film, demonstrating its homogeneity and ensuring consistent results across multiple regions. This comprehensive approach allowed us to address how substrate-induced strain and sample homogeneity influence the observed SMR and SSE behavior.

### 2.2. Methodology of the spin Hall magnetoresistance and spin Seebeck measurements

The Spin Hall magnetoresistance (SMR) and Spin Seebeck effect (SSE) are detected simultaneously with two different techniques: lock-in detection [24] and switching scheme [25]. In the lock-in detection technique, the first and second harmonic voltage responses were recorded separately using lock-in amplifiers in both longitudinal and transverse geometries for CCO/MAO samples (see Figure 1(e)). Additionally, angular-dependent first and second harmonic voltage responses were recorded in the transverse geometry for the CCO/MAO sample in the zx and zy planes (results provided in Appendices B and C). For the CCO/MgO sample, the first and second harmonic voltage responses were recorded only in the transverse geometry (see Figure 1(f)).

The SMR signals were detected as the first-harmonic resistance response, while the current-induced SSE was detected in the second-harmonic resistance response of the Pt Hall bar. To measure SMR, an AC current ($I_{rms}$ = 2–5 mA and f = 17 hz) was passed through a Pt Hall bar and voltage signals were recorded in both longitudinal and transverse geometries. The SMR and SSE were measured at various magnetic fields (0–10 T), and for temperatures ranging from 5 to 300 K. The angle dependence was measured by rotating the external magnetic field in the x-y plane of the film as a function of the angle α, where α is the relative angle between the current direction and the magnetic field H along the x-axis, as introduced in the coordinate system in Figure 1(e). To record the angle dependence of transverse signals in a 40 nm CCO/MAO sample, we used the switching scheme Ref [25], in which the voltage signals were recorded using a Keithley 2182 nanovoltmeter. The SMR and SSE signals were separated by (V(+J)−V(−J))/2 and (V(+J)+V(−J))/2, respectively. Here, J represents the current density through the Pt Hall bar. After separating the SMR and SSE contributions, the data is processed similarly to the lock-in detection technique.

**Figure 2.**
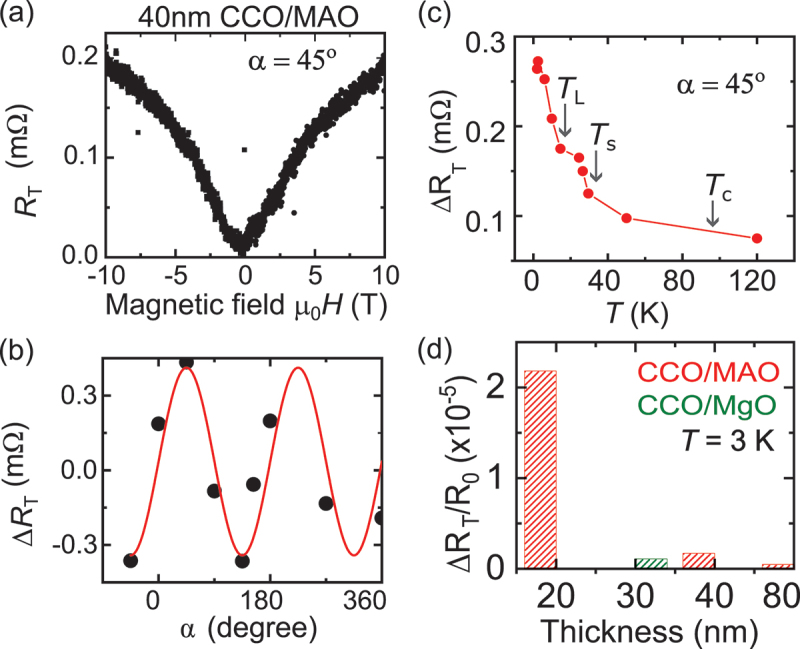
(a) Magnetic field dependence of the transverse Pt resistance R${\;}_{T}$ at 5K for $\alpha =45^{\circ }$ on a 40 nm thick CCO/MAO sample. (b) Angle dependence of $\Delta R_{T}$ fitted with a sin2α function (red curve). $\Delta R_{T}$ represents the maximum variation in transverse magnetoresistance, calculated as [$R_{T}(\mu_{0}H=\pm 10T)-R_{T}(\mu_{0}H=0T)$]. (c) Temperature dependence of $\Delta R_{T}$, illustrating changes in magnetoresistance with varying temperature. (d) SMR ratio ($\Delta R_{T}/R_{0}$) as a function of film thickness, where $R_{0}$ denotes the longitudinal Pt resistance at $\alpha =0^{\circ }$.

We employed two distinct approaches to investigate the changes in SMR around the spin-spiral and spin lock-in phases. In the first approach (see Figure 2), we measured the transverse SMR signals ($R_{T}$) by maintaining a constant magnetic field angle (α) while varying its strength, similar to recording a magnetization hysteresis loop. These $R_{T}$-versus-field curves were then used to analyze the transverse SMR’s dependence on the magnetic field angle (α), temperature, and film thickness.

**Figure 3.**
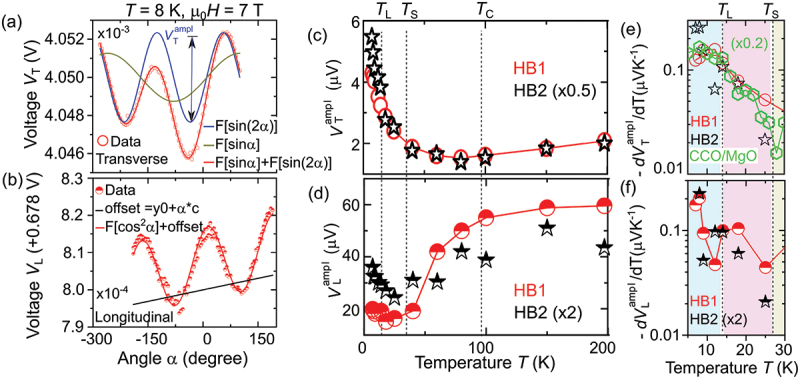
Angular dependence of (a) transverse and (b) longitudinal first harmonic response of Pt electrode. Panels (c) and (d) display the temperature dependence of the amplitude of spin Hall magnetoresistance for two Hall bar devices (HB1 and HB2) on a 40 nm thick CCO/MAO film, illustrating transverse and longitudinal geometries, respectively. $V_{T}^{ampl}$ $V_{L}^{ampl}$ are defined as the amplitude of sin(2α) function for transverse magnetoresistance and for longitudinal magnetoresistance, respectively. Panels (e,f) demonstrate the temperature derivative of magnetoresistance shown in 3(c) and 3(d).

In the second approach, we determined the SMR amplitude by fitting the SMR voltage signals obtained as a function of angle α in both longitudinal and transverse geometries (Figure 3(a,b))). This allowed us to gain insight into the SMR response at varying temperatures, especially around critical magnetic phase transitions such as spin spiral, at $T_{S}$, and spin lock-in phase transition at $T_{L}$. The SMR amplitude $V_{T}^{ampl}$ for the transverse geometry was obtained by fitting the transverse voltage signal $V_{T}$ with $V_{T}=V_{0}+V_{T}^{ampl}\sin2(\alpha -\phi )+V_{H}\sin\alpha$ (see Figure 3(a)), where $V_{H}$ represents the contribution originating from the ordinary Hall effect. Note that a sin2α dependence is observed with a phase shift ϕ of 5${\;}^{\circ }$ to 30${\;}^{\circ }$ at 7T as a function of temperature. This phase ϕ can be related to surface twists at the interface, as reported in Ref [6]. To obtain $V_{L}^{ampl}$ in longitudinal geometry, we fitted the longitudinal voltage signal $V_{L}$ with the $V_{L}=V_{0}+\mathrm{c\alpha}+V_{L}^{ampl}\cos^{2}(\alpha -\phi )$ equation (see Figure 3(b)), where cα accounts for thermal drifts which are approximated as contributing linear in time or linear in α. The contribution cα could also arise from the magnetoresistance of Pt if it becomes magnetic when in close proximity to the COCr${\;}_{2}$O${\;}_{4}$ magnet. To rule out any proximity-induced effects in Pt, we conducted x-ray magnetic circular dichroism measurements, which are discussed later.

**Figure 4.**
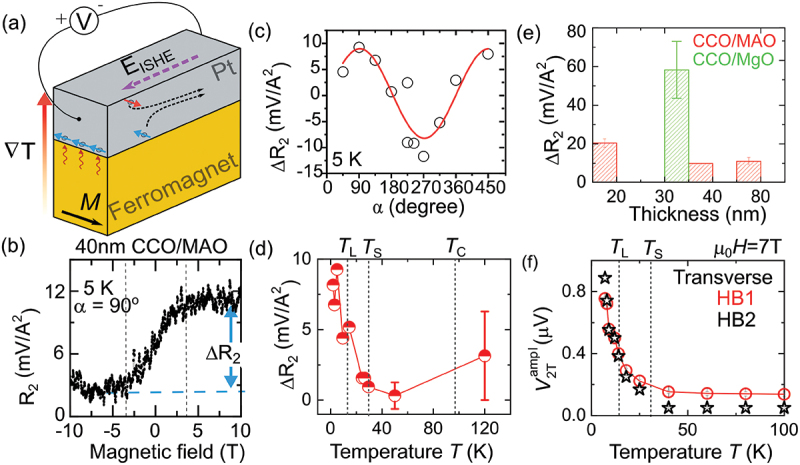
(a) Schematic illustration of the longitudinal spin Seebeck effect: a thermal gradient across the ferromagnet induces a spin accumulation in Pt, detectable as an inverse spin Hall voltage. (b) Second harmonic Pt resistance R${\;}_{2}$ as a function of the magnetic field at α = 90${\;}^{\circ }$ for a 40 nm thick CCO/MAO sample. The spin Seebeck signal $\Delta R_{2}$ is defined as the maximum change in resistance response [$R_{2}(\mu_{0}H=\pm 8T)-R_{2}(\mu_{0}H=0T)$]. (c) Variation of $\Delta R_{2}$ as a function of angle α at 5 K for a CCO/MAO sample. (d) Temperature dependence of $\Delta R_{2}$, highlighting changes in the spin Seebeck signal with temperature. (e) Relationship between film thickness and spin Seebeck signal $\Delta R_{2}$ at 5 K measured at α = 90${\;}^{\circ }$ in CoCr${\;}_{2}$O${\;}_{4}$ films. (f) Temperature dependence of the spin Seebeck amplitude $V_{2T}^{ampl}$ for the transverse Hall bar geometry. $V_{2T}^{ampl}$ is derived from the in-plane angular dependence of second harmonic Pt voltage response V${\;}_{2T}$ in transverse geometry.

To analyze the trend of the SSE, we employed two approaches similar to those used for the SMR signals. In the first approach, we measured the hysteresis loops of the SSE signals and used these loops to examine further the dependence of SSE on angle α, temperature, and thickness of the film (see Figures 4(a–e)). To determine the SSE amplitude, we fitted the angle-dependent SSE voltage signals using equations $V_{2T}=V_{2T}^{0}+V_{2T}^{ampl}\cos(\alpha )$ for transverse geometry and $V_{2L}=V_{2L}^{0}+V_{2L}^{ampl}\sin(\alpha )$ for longitudinal geometry. In these equations, $V_{2T}^{0}$ and $V_{2L}^{0}$ represent angle-independent offsets observed in the Pt second harmonic response. Figure 4(f) shows an example of the SSE amplitude obtained by fitting the angular-dependent SSE voltage in the transverse geometry, measured at various temperatures.

### 2.3. X-ray magnetic circular dichroism

X-ray magnetic circular dichroism (XMCD) measurements were performed at 2 K on the BOREAS beamline of the ALBA synchrotron light source [26]. XMCD spectra were obtained by calculating the difference between right ($\mu_{+}$) and left ($\mu_{-}$) x-ray absorption spectra of circularly polarized light with 70% and 100% polarization for the Pt *M*- and Co or Cr L-edges, respectively. During XMCD, a magnetic field up to 6 T was applied either in-plane at grazing incidence (30${\;}^{\circ }$ from the film plane) or out-of-plane along the 001 direction at normal incidence, maintaining a pressure of up to 10${\;}^{-10}$mbar at the sample place.

## 3. Results

### 3.1. Spin Hall magnetoresistance

We measure the temperature dependence of the spin Hall magnetoresistance (SMR) to electrically detect changes in magnetic states of CCO samples.SMR, as theoretically [27,28] and experimentally [1–3,6,7,24] demonstrated, is a phenomenon where the resistance of a metallic or semiconducting electrode, in our case platinum, changes due to the reflection or absorption of spin-polarized charge carriers due to the spin Hall effect, dependent on the magnetization direction of a ferromagnetic insulator (FM) underneath. For the case that the magnetization is parallel to the polarization direction of the charge carriers in the spin accumulation region of the metal, the spin current gets reflected back into the Pt electrode, leading to an additional charge current through the inverse spin Hall effect (ISHE). In contrast, when the magnetization direction is oriented perpendicular to the spin polarization in the accumulation layer, an effectively increased resistivity is observed due to the ability to have a finite spin transfer torque leading to additional dissipation in the Pt electrode. The longitudinal resistance establishes a $\cos^{2}\alpha$ dependence with the maximum modulation at α = 0${\;}^{\circ }$ or 90${\;}^{\circ }$ where the transverse SMR (planar Hall effect) vanishes. The transverse SMR has a sin2α dependence with a maximum at α = 45${\;}^{\circ }$.

**Figure A1.**
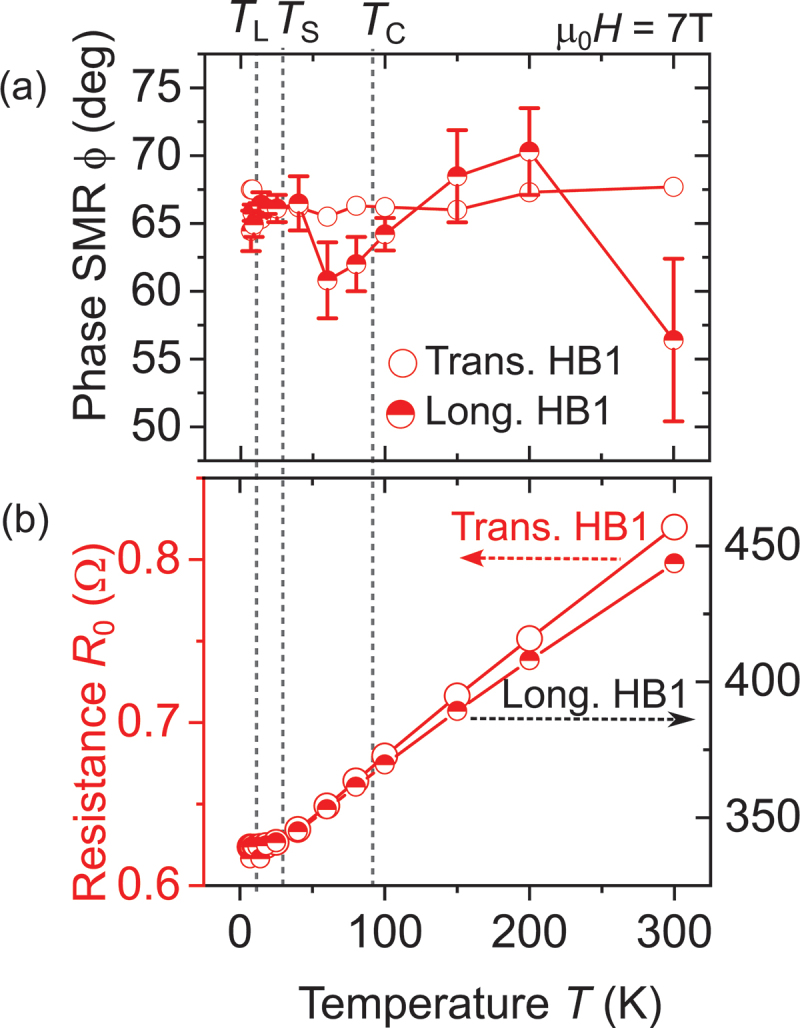
(a) Temperature dependence of the phase ϕ in both longitudinal and transverse directions. The phase ϕ is determined directly by fitting the spin-Hall magnetoresistance signal measured at a constant temperature as a function of the in-plane applied magnetic field angle α. (b) Shows the temperature dependence of the α-independent resistance of the Pt Hall bar.

**Figure B1.**
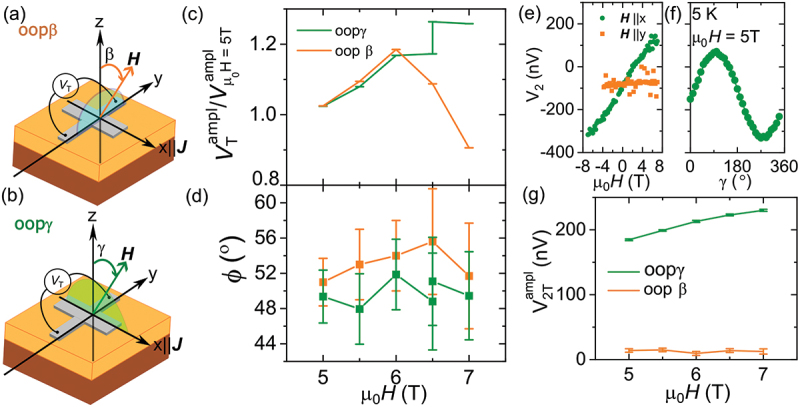
(a) and (b) Schematics of two rotational planes (oopβ and oopα) with respect to the [001] out-of-plane film axis. (c) and (d) the normalized transverse spin Hall magnetoresistance amplitude ($V_{T}^{ampl}/V_{\mu_{0}H=5T}^{a\mathrm{mpl}}$) and the phase ϕ are plotted as a function of the magnetic field, derived from angular-dependent measurements in the oopβ and oopα planes at 5 K. (e) and (f) Magnetic field and angular dependence of the second harmonic Pt resistance response. (g) Magnetic field dependence of the spin Seebeck amplitude $V_{2T}^{ampl}$ extracted from the angular dependence of the second harmonic voltage signal measured in the transverse configuration at 5 K.

The transverse SMR, $R_{T}$, was defined as $V_{T}/I$ (Figure 2(a)), where $V_{T}$ is the first harmonic transverse voltage, exhibiting a dip/peak as a function of the applied magnetic field at fixed values of α in 40 nm thick CCO/MAO samples, agreeing well with previous findings in the Pt/YIG system [2]. Figure 2(a) shows the transverse SMR $R_{T}$ after subtracting the ordinary Hall effect contribution due to a slight sample misalignment (see Figure B1 to compare the ordinary Hall effect contribution). For α = 180${\;}^{\circ }$, $R_{T}$ saturates ($\mu_{0}H\geq$ 6T for one field direction), indicating that the applied in-plane magnetic field is larger than the anisotropy fields for the CCO/MAO sample. The maximum change in the SMR signal $\Delta R_{T}$ is defined at $\mu_{0}H=10T$ w.r.t to $R_{T}$ observed at zero applied magnetic field. Figure 2(b) shows a sin2α dependence of $\Delta R_{T}$ signal as expected for the transverse SMR signal. The temperature dependence of $\Delta R_{T}$ increases as the temperature decreases, displaying distinct anomalies that highlight the spiral transition temperatures, $T_{S}$ and $T_{L}$, as shown in Figure 2(c). The ratio $\Delta R_{T}/R_{0}$ increases as the thickness of the CCO film decreases, with a 20 nm thick CCO/MAO sample exhibiting five times larger ratio than a 30 nm thick CCO/MgO sample where $R_{0}$ is the longitudinal Pt resistance at α = 0${\;}^{\circ }$ (see Figure 2(d)). Importantly, the measured resistance $R_{0}$ does not show any jumps in the expected temperature range of noncollinear phases of CCO below 100K (see Figure A1(b)).

Figures 3(c,d) display the extracted SMR amplitude ($V_{T}^{ampl}$ and $V_{L}^{ampl}$) obtained from the angular dependence of SMR for two separate Hall bar devices on a CCO/MAO sample, as demonstrated in Figures 3(a,b) for transverse and longitudinal geometries, respectively. A non-zero SMR signal persists in the paramagnetic phase above the ferrimagnetic transition temperature $T_{c}$ up to room temperature for both transverse and longitudinal geometries, in line with previous observations [7]. Below $T_{c}$, an exponential increase of the transverse SMR $V_{T}^{ampl}$ signal is observed with decreasing temperature, resulting in a linear increase in $dV_{T}^{ampl}/dT$ in a logarithmic plot as shown in Figure 3(e). The temperature dependence of $V_{T}^{ampl}$ reveals an almost one-order of magnitude increase in the SMR signal within the spin lock-in phase compared to that observed at $T_{C}$, as illustrated in Figure 3(c). Contrary to the $V_{T}^{ampl}$, the longitudinal SMR $V_{L}^{ampl}$ decreases by reducing the temperature below $T_{c}$ until $T\approx T_{S}$ (cf. Figures 3(c,d), resulting in a clear slope change at $T\approx T_{L}$ as evident from $dV_{L}^{ampl}/dT$ (cf Figures 3(e,f)). Further, a decrease in temperature below $T_{S}$ results in an increase in the $V_{L}^{ampl}$ for decreasing temperatures.

Below the transition temperature ($T_{S}$), the CCO film exhibits a spin-spiral magnetic ordering state, consisting of a collinear component contributing to the net magnetization and a spiral component lying in a plane perpendicular to the collinear magnetization. The relative contributions of these components are governed by the conicity (ξ) of the spin ordering, defined as the ratio of the collinear to spiral components of the magnetization:

(1) $$\xi =\frac{M_{\mathrm{collinear}}}{M_{\mathrm{spiral}}}=\cot\theta$$

Here, $M_{co\mathrm{llinear}}$ and $M_{sp\mathrm{iral}}$ are the collinear and spiral components of magnetization, respectively, and θ is the cone angle. A small cone angle $\theta \ll 45^{\circ }$ corresponds to large conicity, where the collinear component dominates, while a larger cone angle $\theta >45^{\circ }$ represents small conicity, where the spiral component dominates. The conicity is influenced by strain-induced effects and antiphase boundaries (APBs) [18], which are regions of localized strain gradients and magnetic disorder generated during film growth. These APBs, more prevalent in thinner films, disrupt surface magnetization coherence and reduce the longitudinal SMR below $T_{C}$, while having little impact on transverse SMR for $T_{S}<T<T_{C}$. Interestingly, in both longitudinal and transverse geometries, SMR signals increase in the spiral phases instead of decreasing.

In collinear magnetic systems, angle-dependent SMR voltage is typically estimated based on the in-plane magnetic moment density at the interface [28] and the projection of the normalized magnetization direction in the respective coordinate system, given by the following equations

(2) $$V_{SMR}^{T}\propto 2m_{x}m_{y}$$

(3) $$V_{SMR}^{L}\propto m_{x}^{2}-m_{y}^{2}$$

where $m_{x}$ and $m_{y}$ represents the in-plane components of the magnetization unit vector m. However, in the spin-spiral state in CCO, the interpretation of the SMR becomes complex due to the coexistence of three conical spirals on A, B${\;}_{1}$ and B${\;}_{2}$ sites with different cone angles [14], as well as by the presence of four domains with the spiral wave vectors along the ±[110] and $\pm [1\overset{ˉ}{1}0]$ directions. In addition, at low applied magnetic fields the orientations of the cone axes are field-dependent, as intrinsic magnetic anisotropies compete with the Zeeman field. However, our XMCD data (see Figures 1(c,d)) suggest that above ∼5 T the cone axes align with the magnetic field in all conical spirals and for all wave vectors. This re-orientation transition, occurring at a much lower field (∼0.2 T) in bulk crystals [17,29], is shifted to higher fields in thin films due to stronger magnetic anisotropy [18,19].

For a single conical spiral with the cone angle θ, the transverse SMR, obtained from Eq. (2), is proportional to $(3\cos^{2}\theta -1)/2$ [3]. It changes sign at $\theta ∼55^{\circ }$, as a result of the cancellation between the positive contribution of the uniform component and the negative contribution of the spiral component, in which spins are orthogonal to the magnetic field. The cone angles of the three spirals, obtained by neutron diffraction [14], are $48^{\circ }$, $71^{\circ }$ and $28^{\circ }$, which strongly reduces the amplitude of the SMR signal compared to the collinear (θ=0) ferrimagnetic state. Yet, at 5 K, we observe a six times larger transverse SMR signal compared to that at $T_{c}$ and this observation is consistent with the trend reported in the CCO/MgO sample [7]. The sharp upturn of the SMR amplitude at low temperatures is unlikely related to temperature-dependent conicity since the magnetic moment ∝cosθ does not show strong T-dependence. We also find a distinctively large phase shift, ϕ, of the SMR signal (see Figure A1(a)) indicative of a misalignment between the magnetization and the applied magnetic field, which may result from a twist of the magnetic structure at the interface due to the *interface* Dzyaloshinskii–Moriya interaction or magnetic anisotropy. Alternatively, ϕ can result from the SMR terms proportional to the magnetization gradient [6,30]. In the conical spiral state of the chiral magnet, Cu${\;}_{2}$OSeO${\;}_{3}$, these terms enhance the transverse SMR signal by a factor of 2 [6]. In the centrosymmetric CCO, the SMR terms linear in the magnetization gradient cancel after averaging over domains with four different orientations of the spiral wave vector. This cancellation can be suppressed by the electric field poling used to measure the electric polarization induced by the spiral component [12,15].

Despite these complexities, the angular dependence of SMR remains sinusoidal even at lower field ∼ 1T, as shown in the Appendix A, suggesting that a dominant magnetization direction exists despite local variations caused by APBs or surface anisotropy. The observed large SMR phase ϕ, which is relatively independent of temperature, indicates significant surface anisotropy and suggests a difference in spiral cone angles at the surface compared to the bulk. This difference allows magnetization gradient terms to remain nonzero at the surface, further enhancing the transverse SMR signal in the spiral phase. The effects of APBs are more pronounced in CCO/MgO films than in CCO/MAO films, as evidenced by the relatively larger SMR phase ϕ observed in the former. Additionally, SMR signals are stronger in thinner films, where surface anisotropy and APBs have greater influence, consistent with the hypothesis of a distinct spiral cone angle at the surface compared to the bulk. Thin CCO films are expected to exhibit significant surface effects due to strain, especially when compared to thicker films. The pronounced increase in SMR at low temperatures is likely a surface-related property, further suggesting that the observed effects may be driven by interface characteristics.

In contrast to the transverse SMR $V_{T}^{ampl}$, a decrease in the longitudinal SMR $V_{L}^{ampl}$ is observed with decreasing temperature in the ferrimagnetic phase ($T_{s}<T<T_{c}$) (cf. Figures 3(c,d)). This difference indicates that the additional higher-order contribution provides the chiral part of the overall signal, which is anisotropic in q. These findings align with theoretical predictions by Kipp and coworkers [30], suggesting anisotropic contributions to the transport properties in spiral magnets. These results show that the Pt/CCO system provides an experimental platform to test theoretical predictions of chiral contributions of SMR. However, full theoretical calculations of these contributions are out of the scope of this study.

### 3.2. Spin seebeck effect

The Spin Seebeck effect (SSE) can be retrieved from the second-harmonic response in the Pt layer. The SSE originates from Joule heating within the Pt Hall bar, generating a heat current directed toward the ferromagnetic insulator. This thermal gradient generates a spin current driven by thermal excitations in the magnetization (magnons) of the ferromagnet. At the interface to a spin Hall system like Pt, this spin current can modify the resistance of the Pt electrode through the inverse spin Hall effect, as sketched in Figure 4(a). The E${\;}_{ISHE}$ signal, generated by the inverse spin Hall effect satisfies the relationship $E_{ISHE}\propto \nabla T\times M_{eq}$ [31,32], where $M_{eq}$ and ∇T represent the equilibrium magnetization close to the interface and the thermal gradient along z-direction, respectively. Therefore, the SSE voltage generated by the $E_{ISHE}$ can be used to unveil the in-plane magnetization orientation and magnetic anisotropies.

The observed second harmonic response $R_{2}=\sqrt{2}V_{2}/I^{2}$ measured at an angle α = 90${\;}^{\circ }$ in transverse resistance shows a clear saturation above ±5T (see Figure 4(b)), where $V_{2}$ denotes the second harmonic voltage measured using a lock-in amplifier. In contrast to $R_{2}$, the magnetometry measurements done by a SQUID magnetometer do not observe a precise magnetization saturation due to significant background contributions from the substrates. Nevertheless, a clear hysteresis loop is observed by XMCD measurements as exemplified in Figure 1(c,d). In contrast to the CCO/MAO sample, the CCO/MgO sample does not exhibit saturation of the transverse SSE ($R_{2}$) with magnetic field, even up to 8T. Studies have demonstrated that CCO films grown on MAO substrates exhibit exceptional quality [18]. Conversely, CCO films on MgO substrates display a less perfect lattice structure, characterized by the presence of antiphase boundaries at the surface [33]. The presence of defect and antiphase boundaries at the surface can affect the SSE and shift the saturation field to higher values as observed earlier for SSE in the presence of surface-induced magnetic anisotropies [8,34,35]. We define the maximum change in $R_{2}$ as $\Delta R_{2}=R_{2}(\mu_{0}H=\pm 8T)-R_{2}(\mu_{0}H=0T)$ measured at fixed values of the angle α. Note that $\Delta R_{2}$ shows the expected sinα angular dependence associated with the SSE, as depicted in Figure 4(c). Similar to SMR, $\Delta R_{2}$ also increases as the temperature decreases, exhibiting distinct anomalies at the transition temperatures $T_{S}$ and $T_{L}$ (Figure 4(d)). $\Delta R_{2}$ is approximately six times larger in CCO/MgO sample compared to that of CCO/MAO (see Figure 4(e)). This larger signal in CCO/MgO sample can be related to strain and surface-induced magnetic anisotropies.

**Figure 5.**
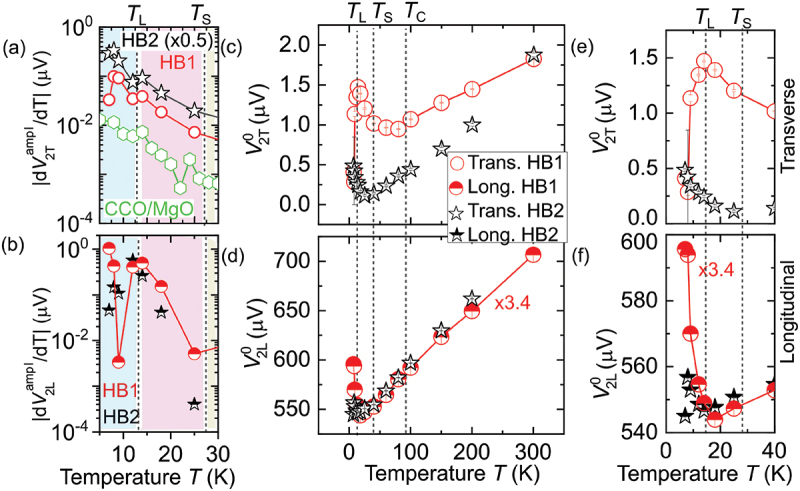
Panels (a) and (b) show the temperature dependence of the derivative of the spin Seebeck amplitudes ($V_{2T}^{ampl}$ and $V_{2L}^{ampl}$) for transverse and longitudinal geometries, respectively. Panels (c-d) depict the temperature-dependent (α independent) spin Seebeck signal. Panels (e–f) show Zoomed-in plots to elaborate data across the spin-spiral ($T_{s}$) and spin lock-in ($T_{L}$) magnetic transition temperatures.

To measure the temperature dependence of the spin Seebeck signal, we subsequently measured the angle-dependent second harmonic Pt voltage response at $\mu_{0}H=7$T at a fixed temperature and extracted the SMR amplitudes $V_{2T}^{ampl}$ and $V_{2L}^{ampl}$ for both transverse and longitudinal geometries, respectively. The temperature-dependent behavior of $V_{2T}^{ampl}$ and $V_{2L}^{ampl}$ exhibits a similar pattern, showing a notable enhancement (close to an order of magnitude) below $T_{L}$ compared to the signal observed at $T_{C}$. The temperature dependence of $V_{2T}^{ampl}$ is exemplified in Figure 4(f). The substantial increase in SSE at lower temperatures for $T<T_{S}$ can be attributed to the temperature dependence of thermal spin conductivity [36] and magnon relaxation times [37]. In the Pt/YIG systems, it has been demonstrated that this relaxation time can be characterized by the interplay of two dominant mechanisms: scattering by defects, primarily active at lower temperatures, and magnon–magnon scatterings, which come into play at elevated temperatures. To confirm this, it will be interesting to check the thermal spin conductivity and magnon relaxation times as a function of temperature in Pt/CCO systems. Nevertheless, Figures 5(a,b) distinctly expose the spin lock-in transition around $T_{L}$ in the temperature-dependent derivatives of the transverse SSE $dV_{2T}^{ampl}/\mathrm{dT}$ and longitudinal SSE $dV_{2L}^{ampl}/\mathrm{dT}$, respectively. Interestingly, α independent contribution to the second order voltage response also strikingly changes near spin lock-in transition temperature $T_{L}$ (see Figs Figure 5(c–f)).

According to the theory of the Spin Seebeck effect (SSE) [38], the SSE voltage signal is proportional to the average in-plane magnetization. For a single conical spiral with a cone angle θ, this means that the SSE should be proportional to cosθ. Therefore, we do not expect a significant increase in the SSE as observed in Figure 4(f). The SSE, in contrast, exhibits an upturn at lower temperatures ($T<T_{S}$). The small changes in the slope of the SSE can be attributed to subtle variations in the conicity of the spiral phases. Above 6 T, when the spin-spiral planes in all three spirals become orthogonal to the applied magnetic field, the dependence of the conical angles on the applied field becomes weak, and magnetization grows very slowly with the field. Similarly, the conical angles are expected to remain largely unchanged with temperature, aside from minor shifts at phase transitions that may result in small anomalies. Consequently, the steep upturn of the SSE and SMR signals at low temperatures cannot be fully explained by changes in the conical angles. Since SSE signals respond only to the uniform component of the conical spiral, we observe an overall increase in SSE as the film thickness increases for CCO/MAO films.

The significant increase in SSE at low temperatures is more likely due to an enhancement in magnon thermal conductivity, similar to what has been observed in Cu${\;}_{2}$OSeO${\;}_{3}$, where the SSE strictly follows the power law of magnon thermal conductivity with a maximum at T=2K [36]. Understanding this pronounced increase in SSE at low temperatures requires a detailed examination of magnon damping and the thermal conductivity of magnons. A complete theoretical explanation for the significant SSE changes observed below $T_{L}$ is beyond the scope of this manuscript.

### 3.3. X-ray magnetic dichroism

X-ray absorption spectra (XAS) are measured through total electron yield on CCO/MAO, utilizing circularly polarized light at normal incidence while a magnetic field of $\mu_{0}H$ = 6 T is applied perpendicularly to the layer. Given the substantial SMR and SSE signals observed at low temperatures, the magnetic proximity effect within the Pt electrode needs to be quantified. The magnetic proximity effect would lead to similar signals.

**Figure 6.**
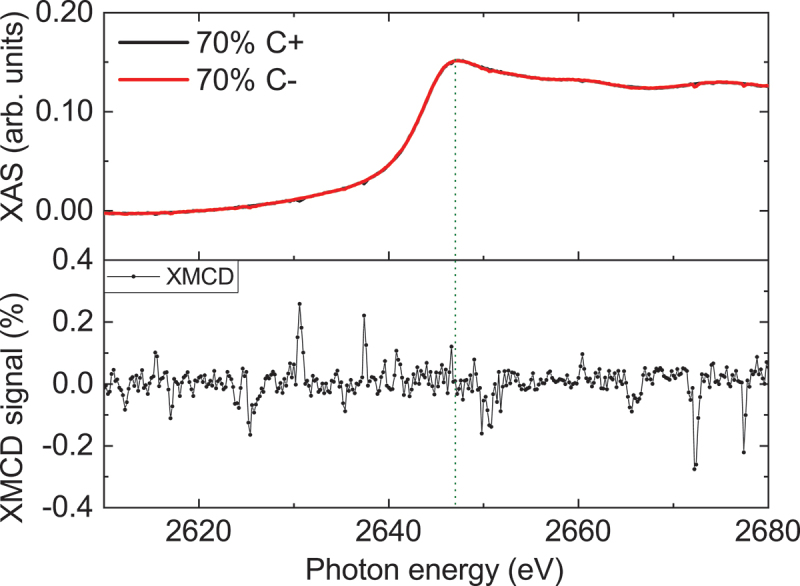
XAS (top) and XMCD (bottom) spectra measured at 2 K at the Pt $L_{3}$ edge for Pt (5 nm)/CoCr${\;}_{2}$O${\;}_{4}$ (40 nm)/MgO heterostructures with $\mu_{0}H$ = 6 T applied along [100] direction perpendicular to the film plane. The dotted line indicates the white line of spectra.

To mitigate contributions from the magnetic proximity effect, we examined the XMCD signals at 2K in 5 nm thick Pt electrodes deposited on the CCO films. The resulting XMCD data demonstrated no discernible magnetic proximity effect, see Figure 6. These findings affirm that, if present, the Pt magnetic moment at the white line of the spectra (indicated by the dotted line) remains below the detection limit (≪0.002 $\mu_{B}$/Pt) [21]. Contributions to the observed SMR and SSE signals by a proximity-induced magnetization in the Pt electrodes can, therefore, be ruled out.

### 3.4. Magnetic ordering and its impact on SMR and SSE

By ruling out any significant proximity-induced magnetization in the Pt layer, as evidenced by the absence of a detectable XMCD signal from Pt, we ensure that the observed SMR and SSE signals are intrinsic to the spin transport phenomena at the Pt/CCO interface. The most prominent feature in the temperature dependence of the SMR and SSE signals is their steep increase at temperatures below $T_{S}$. This common trend must be related to the increase of the coherence length of the incommensurate magnetic ordering that shows a similar temperature dependence [14]. In the ferromagnetic phase ($T_{S}<T<T_{C}$), the uniform component of the conical spiral ordering is long-ranged, whereas the spiral component has a relatively short correlation length. This fluctuating spiral ordering gives rise to a disorder that shortens magnon lifetime. Below $T_{S}$, the multiple conical spiral ordering sets in and both the uniform and incommensurate spin-spiral orders are nominally long-ranged, although neutron diffraction data suggest that the correlation length of the incommensurate ordering remains finite and keeps growing with decreasing T even below $T_{S}$ [14]. The decrease in disorder enhances the magnon lifetime, thermal spin conductivity, and the spin current through the interface [36,39,40], which increases the SSE and SMR signals.

In addition, we observe small anomalies at the magnetic transition temperatures related to changes in the spin ordering. They can have various sources, such as the contribution of the spiral component of the conical spiral ordering to the SMR signal that depends on the conical angle [3,41] and the spiral wave vector [6]. The latter effect, not present in uniform ferromagnets, can play an important role in CoCr${\;}_{2}$O${\;}_{4}$ with a short spiral wavelength. In addition, the presence of three conical spirals in CoCr${\;}_{2}$O${\;}_{4}$, one formed by Co spins on A sites and two spirals formed by Cr spins on B1 and B2 sites may play an important role. These three spirals have different conical angles and their relative contributions to the SSE and SMR effects may change across the phase transitions. The anomalies at $T_{L}$ might have the same physical origin as the reversal of the magnetically-induced electric polarization observed at the lock-in temperature, which was attributed to the opposite signs of the polarizations induced by Co and Cr spins [15]. Despite the complexities related to the magnetic order, our results underscore the efficient conversion of thermal magnons to an ISHE signal at low temperatures. These observations are consistent with the magnon-driven nature of SSE and the interface-driven sensitivity of SMR.

## 4. Conclusions

The main outcome of this study is the successful differentiation of non-collinear magnetic phases in the magnetic insulator CoCr${\;}_{2}$O${\;}_{4}$(CCO) thin films through all-electrical detection. This was accomplished by analyzing the longitudinal and transverse resistance variations of a Pt film in contact with CCO across different temperatures and applied magnetic fields. These variations were interpreted in terms of spin Hall magnetoresistance (SMR) and the spin Seebeck effect (SSE), which revealed phase transitions corresponding to the spin spiral and spin lock-in phases at the expected temperatures. Importantly, x-ray magnetic circular dichroism (XMCD) measurements confirmed the absence of proximity-induced magnetization in the Pt layer, demonstrating that a single Pt layer can reliably detect distinct non-collinear magnetic phases through its contributions to the SMR and SSE.

The similarity between the bulk and thin film transition temperatures suggests that the magnetic states in both cases are fundamentally the same. However, the films require a much stronger magnetic field to align the conical spirals with the applied field, which is consistent with the stronger magnetic anisotropy previously reported for films. Despite this similarity, the temperature dependence of the SMR and SSE amplitudes is difficult to explain in terms of the temperature dependence of the conical angle θ. While the amplitudes of both longitudinal and transverse SMR should be proportional to $(3\cos^{2}\theta -1)/2$, they behave differently between $T_{c}$ and $T_{L}$. Furthermore, the SSE signal, which depends on the conical angle θ differently (being proportional to cosθ or the average in-plane magnetization), exhibits a similar upturn at low temperatures as the SMR signal. A possible explanation for these observations is the increased coherence length of the incommensurate magnetic ordering at low temperatures. The observation of large phase ϕ indicates that magnetic ordering at the interface of thin film differs from that in the bulk. Nevertheless, both SMR and SSE amplitudes display anomalies at phase transitions observed in the bulk material, which is a remarkable finding. Our findings clearly show that a single Pt layer can effectively act as a detector to distinguish between different non-collinear magnetic phases using spin Hall magnetoresistance and the spin Seebeck effect.

## Figures and Tables

**Figure A2.**
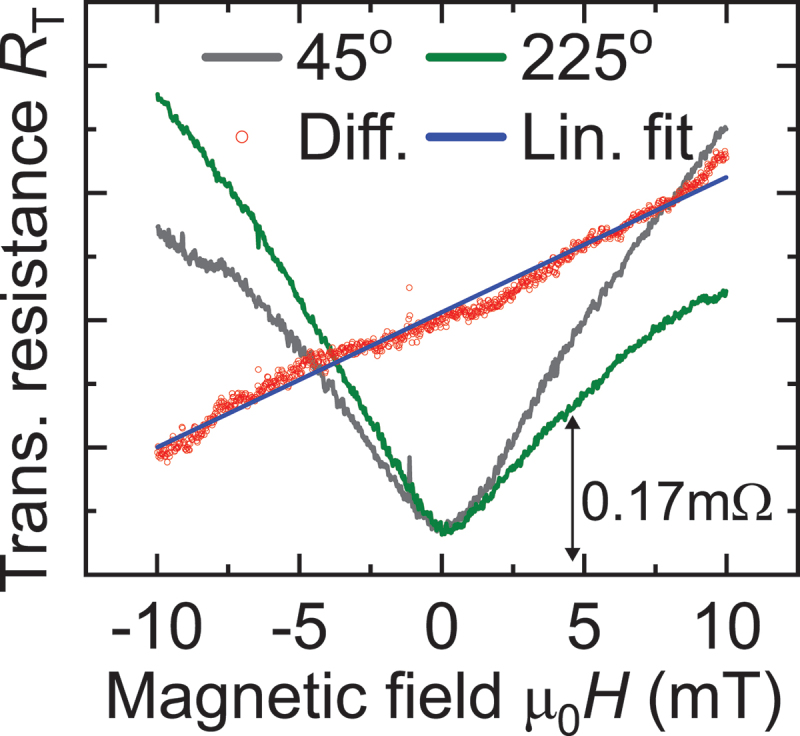
Magnetic field dependence of $R_{T}$ for $\alpha =45^{\circ }$ and $\alpha =225^{\circ }$, where SMR signal is expected to have the same sign. Ordinary Hall contribution obtained as $\left[R_{T}(\alpha =225^{\circ })-R_{T}(\alpha =45^{\circ })\right]$.

**Figure A3.**
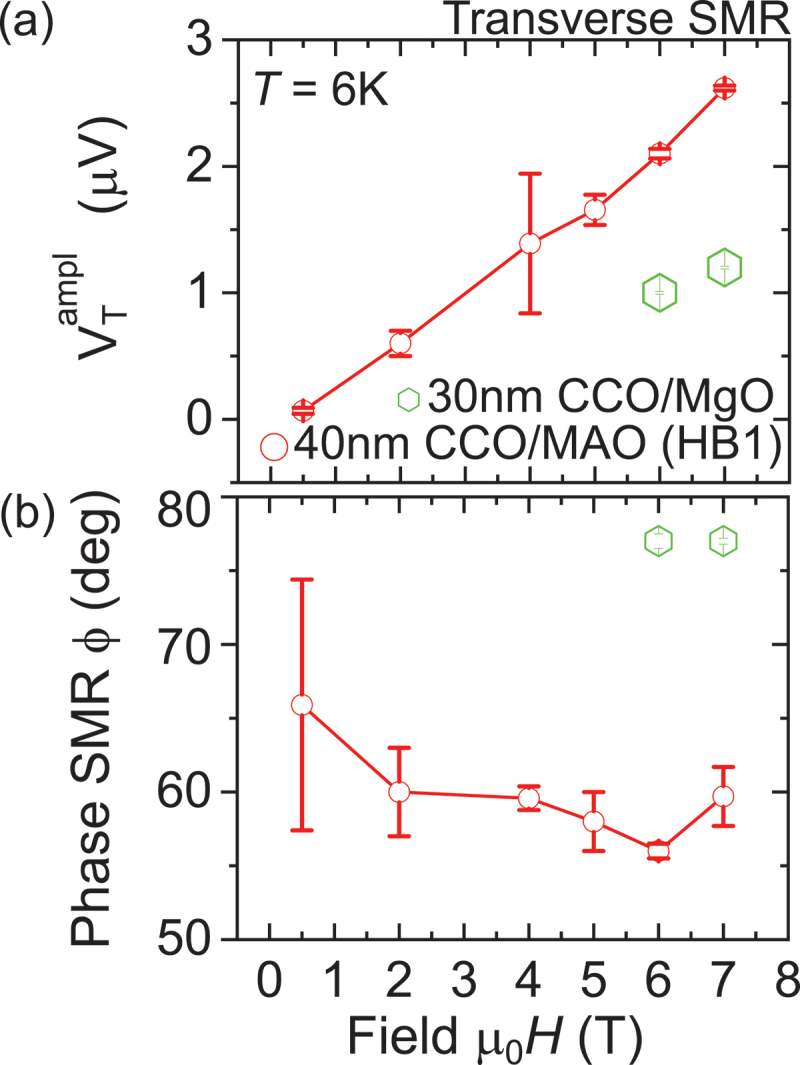
(a) and (b) Field dependence of the transverse spin Hall magnetoresistance amplitude $V_{T}^{ampl}$ and phase ϕ measured at 6 K. The phase ϕ is determined directly by fitting the spin-Hall magnetoresistance signal measured as a function of the in-plane applied magnetic field angle α.

## Data Availability

A data availability statement is compulsory for all research articles.

## Appendix A. Spin-Hall magnetoresistance in xy-plane

To measure the spin-Hall magnetoresistance (SMR), we mainly used lock-in detection [24] and switching scheme [25] techniques as explained in the main text. We mainly performed the SMR measurements in the planar Hall geometry in the xy-plane. Figure A1(a) shows the phase ϕ obtained from the angular dependence of the SMR data, as exemplified in Figure 3(a,b) of the main text. Figure A1(b) shows the angle independent resistance $R_{0}=V_{0}/I$. The resistance $R_{0}$ is measured simultaneously with the phase ϕ. Note that the measured resistance $R_{0}$ does not show any jumps in the expected temperature range of noncollinear phases of CCO below 100K (see Figure A1(b)). In the transverse resistance, an additional contribution $V_{H}$ with sine periodicity is observed along with the expected SMR signals. This $V_{H}$ contribution is associated with the ordinary Hall effect, caused by a slight misalignment of the sample relative to the applied magnetic field. As expected for the ordinary Hall effect, $V_{H}$ scales linearly with the applied magnetic field, as shown in Figure A2.

## Appendix B. Spin-Hall magnetoresistance in zy- and zx-planes

In addition to the xy-plane analysis presented in the main text, we performed angular-dependent spin-Hall magnetoresistance (SMR) measurements in the zy- and zx- planes, referred to as oopβ and oopγ, respectively (see Figure B1(a,b)). The ordinary Hall effect contributions are relatively large in the zx and zy planes which makes it challenging to isolate the transverse SMR signals from the total first harmonic voltage signals. Nevertheless, the extracted SMR data from these out-of-plane configurations complement the in-plane results and exhibit consistent trends. Specifically, the normalized transverse SMR amplitude ($V_{T}^{ampl}/V_{\mu_{0}H=5T}^{a\mathrm{mpl}}$) increases with the magnetic field, following the same trend observed in the xy-plane till 6T (see Figure A3(a)). Beyond 6 T, the SMR signal starts to saturate in the zx plane while a decrease in the SMR signal is observed in the yz plane (see Figure B1(c)). Despite these variations, for all field scans with $\mu_{0}H\geq$1T, a sinusoidal angular dependence of the SMR signal is consistently observed. These results provide further insights into the anisotropic SMR behavior and the influence of out-of-plane magnetization components in the Pt/CCO system. Similar to the xy-plane, a distinct SMR phase ϕ is observed in the zy- and zx-planes, which does not exhibit a strong dependence on the magnetic field strength (cf. Figures A3(b)) and B1(d)).

## Appendix C. Spin Seebeck effect in zy- and zx-planes

Similar to the xy-plane, the transverse spin Seebeck effect (SSE) measurements in the zy- and zx-planes also reveal a consistent trend. As expected, the transverse SSE signal is nearly zero when the magnetic field is fully aligned along the y-direction (see Figure B1(e)). A finite SSE signal is observed when the field is aligned along the x-direction, demonstrating the expected sinγ periodicity in the yx-plane, as shown in Figure B1(f). This behavior is consistent with the symmetry and orientation of the spin Seebeck effect in these out-of-plane configurations. The SSE amplitude, obtained from the angular dependence of the SSE in the zx-plane, shows an increase with the applied magnetic field (see Figure B1(g)). While a zero SSE signal is theoretically expected in the zy-plane, a finite but very small and field-independent signal is observed in this configuration.
